# Hematology research output from Chinese authors and other countries: a 10-year survey of the literature

**DOI:** 10.1186/s13045-014-0103-3

**Published:** 2015-02-06

**Authors:** Lei Zhang, Xin Ye, Yi Sun, An-mei Deng, Bao-hua Qian

**Affiliations:** Department of Laboratory Diagnosis, Changhai hospital, Second Military Medical University, Shanghai, 200433 China; Department of Transfusion Medicine, Changhai hospital, Second Military Medical University, Shanghai, 200433 China

**Keywords:** Hematology, Publications, Impact factor, Citations, Science Citation Index Expanded (SCIE)

## Abstract

**Background:**

Hematologic disease affects people of all ages worldwide. In the past decade, researchers have made great progress in the field of hematology. In the present study we compared the hematology research output from China and other countries (USA, Germany, UK, Japan and South Korea) over the past 10 years and 5 years.

**Methods:**

The related articles were extracted based on the PubMed database. We recorded the number of publications, clinical trials, randomized controlled trials, meta-analyses, case reports, reviews, citations, impact factors, articles in the top 10 journals and most published journals to assess the quantity and quality of research output in each region.

**Results:**

A total of 120,641 hematology-related articles were published from 2004 to 2013. The USA accounted for 27.13% (32,732/120,641) of the publications, followed by Germany (7,479/120,641; 6.20%), Japan (6,347/120,641; 5.26%), the UK (5,453/120,641; 4.52%), China (2,924/120,641; 2.42%) and South Korea (1,413/120,641; 1.17%). The ranking for cumulative impact factors was as follows: USA; Germany; UK; Japan; China and South Korea. The median impact factors in the UK, USA, and Germany were higher than Japan, South Korea, and China. Interestingly, the median impact factors in the three Asia countries were similar both in 2004–2013 and 2009–2013. The UK had the highest percentage of publications in the top 25% of journals, while China lagged behind and ranked last. When comparing the number of articles in the top 10 journals, the results were similar to the IF findings. Germany had the highest number of average citations, while China had the lowest number of average citation. The status of hematology research output from the 6 countries in 2009–2013 had little difference from 2004–2013.

**Conclusions:**

Thus, the USA has had a dominant role in hematologic research in the past 10 years. Overall, the quality of publications in European countries was better than Asia countries. Although China has made considerable progress in hematology research, the quality of research needs improvement.

**Electronic supplementary material:**

The online version of this article (doi:10.1186/s13045-014-0103-3) contains supplementary material, which is available to authorized users.

## Background

Hematologic disease, including disorders of leukocytes, erythrocytes, platelets, hemostatic mechanisms, vascular biology, immunology, and hematologic oncology, affects the health of people worldwide [1]. In the past decades, the health status of the Chinese population has greatly improved [2]; however, China still has a heavy burden of hematologic disease according to the *2013 China Health Statistics Yearbook*, which was published by the National Health and Family Planning Commission of The People's Republic of China [3].

Generally speaking, the number of published scientific papers represents a country's scientific strength. Similarly, the number of articles in a specific field also represents the level of concern in the field. According to the *Statistical Data of Chinese S&T Papers 2013*, which was released by the Chinese Institute of Scientific and Technical Information, the number of Chinese scientific papers in SCI ranked second amongst the top five countries; the other four countries (in order) were the USA, Germany, Japan, and the UK [4]. Currently, there are no data involving scientific publications by Chinese scholars in the field of hematology. In the present study we determined the contributions made by Chinese authors in the field of hematology between 2004 and 2013. Due to the different systems and funding mechanisms which exist in mainland China, Hong Kong, and Taiwan, articles from Hong Kong and Taiwan were not analyzed. Use of “China” herein referred to mainland China. The comparison of hematology research output among China, the USA, UK, Germany, Japan, and South Korea was not limited to 2004–2013, but also included 2009–2013.

## Results

### Total number and share of articles

**Figure 1:**
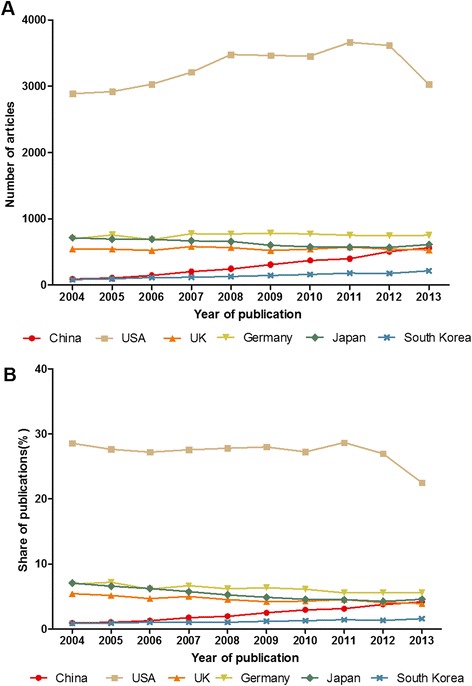
**The number (A) and share (B) of articles published in hematology journals from the six countries (2004–2013).**

A total of 120,641 articles were published between 2004 and 2013 in the 68 hematology-related journals. The USA accounted for the largest proportion (32,732/120,641 [27.13%]), followed by Germany (7,479/120,641 [6.20%]), Japan (6,347/120,641 [5.26%]), the UK (5,453/120,641 [4.52%]), China (2,924/120,641 [2.42%]) and South Korea (1,413/120,641 [1.17%]). The changes in the annual number and share of articles from each country are shown in Figure 1. As shown in Figure 1, the number of papers published from China surpassed the number of papers published from UK in 2013 for the first time, and approached the number of papers published from Japan.

### Clinical trials, randomized controlled trials, case reports, meta-analyses, and reviews

**Figure 2:**
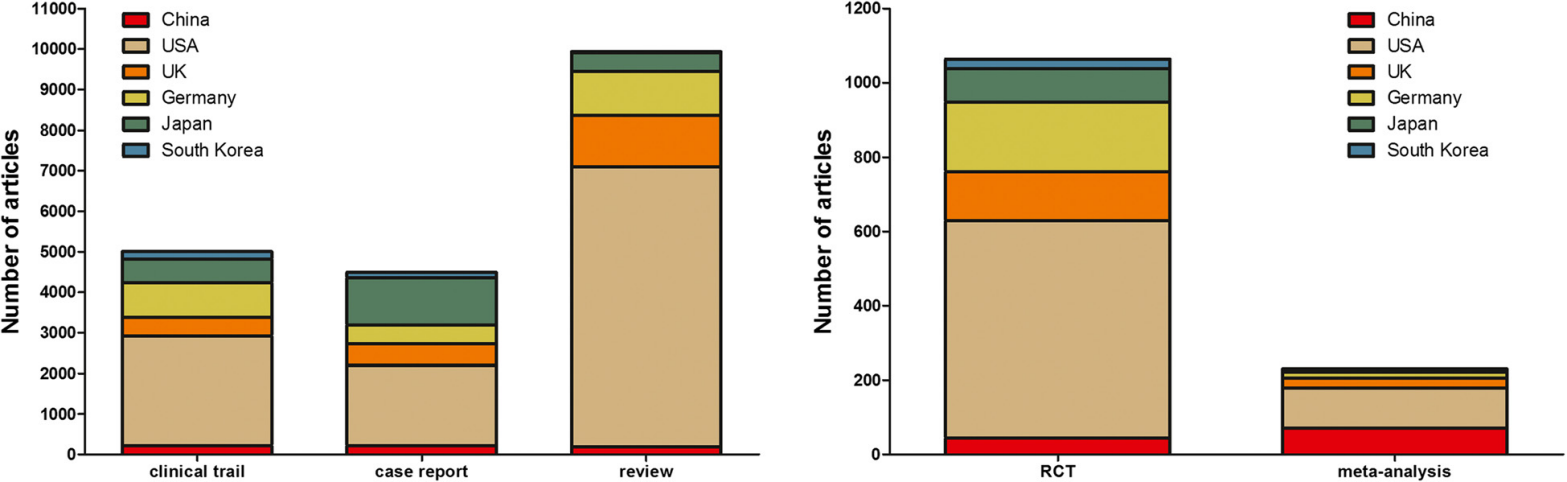
**The number of articles of different publication types from the six countries between 2004 and 2013.**

The number of different types of articles from the six countries is shown in Figure 2. USA ranked first in the number of each type of article. Interestingly, the number of meta-analyses published by China ranked second, followed closely by the USA; other types of publications from China just exceeded the number published from South Korea.

### Impact factor

Based on *JCR 2014*, we calculated the cumulative and median IFs for each geographic region, which are shown in Table 1. The ranking of the cumulative IFs in six countries was the USA, Germany, the UK, Japan, China and South Korea. The UK, USA, and Germany ranked in the top three of median IFs, and the median IFs were equal in the three Asia countries; these rankings did not change in the past 5 or 10 years.

**Table 1:** **Cumulative and median impact factor of articles from each region**

**Year**	**Cumulative impact factors**						**Median impact factors**					
	**China**	**USA**	**UK**	**Germany**	**Japan**	**South Korea**	**China**	**USA**	**UK**	**Germany**	**Japan**	**South Korea**
2004	344.604	15393.468	2515.639	3332.788	2957.428	288.41	2.692	4.202	4.304	3.568	2.806	3.326
2005	387.774	15748.680	2527.391	3869.547	2909.071	415.235	2.692	4.304	4.959	4.421	3.348	2.732
2006	542.250	15728.758	2337.716	3430.667	2705.429	450.98	2.692	4.202	4.304	3.693	2.732	2.692
2007	745.716	16976.891	2777.760	4017.565	2637.568	457.462	2.692	4.304	4.304	4.304	2.732	3.348
2008	872.829	18383.301	2516.288	3528.121	2445.896	460.531	2.732	4.202	4.202	3.466	2.692	2.659
2009	1008.688	18934.461	2272.255	3663.200	2299.502	601.775	2.605	4.304	4.053	3.568	2.692	2.916
2010	1212.098	18158.917	2637.687	3488.621	2209.841	566.511	2.605	3.693	4.959	3.466	2.562	2.562
2011	1398.403	19753.856	2685.009	3638.619	2180.398	619.563	2.692	4.202	4.304	3.568	2.627	2.627
2012	1738.380	18695.207	2454.146	3492.870	2082.804	539.097	2.605	3.568	4.304	3.466	2.562	2.562
2013	1822.023	14523.517	2395.126	3468.483	2048.689	662.632	2.627	3.568	4.959	3.477	2.462	2.468
Total(2009–2013)	7179.592	90065.958	12444.223	17751.793	10821.234	2989.578	2.605	4.053	4.959	3.477	2.605	2.605
Total(2004–2013)	10072.765	172297.056	25119.017	35930.481	24476.626	5062.196	2.692	4.053	4.538	3.568	2.692	2.692

Furthermore, according to the IF, we divided the 68 journals into 4 levels. The number and percentage of publications from each region in the four levels is shown in Tables 2 and 3. From 2004 to 2013 [Table 2], The UK ranked first, while China ranked last with respect to the number of publications in the top 25% of journals (highest level). The same result was demonstrated in the period between 2009 and 2013 [Table 3]. The distribution of publications from each country in the four grades differed little between 2004–2013 and 2009–2013.

**Table 2:** **Distribution of publications from each region in the four grades (2004–2013)**

**Rank**	**IF range**	**China (%)**	**USA (%)**	**UK (%)**	**Germany (%)**	**Japan (%)**	**South Korea (%)**
Top 25%	11.089-4.046	809(27.67)	16901(51.63)	2973(54.52)	3583(47.91)	2170(34.19)	426(30.15)
Top 25%-50%	3.693-2.462	942(32.22)	10815(33.04)	1383(25.36)	1951(26.09)	1486(23.41)	415(29.37)
Bottom25%-50%	2.427-1.679	770(26.33)	3204(9.79)	598(10.97)	1310(17.52)	1726(27.19)	376(26.61)
Bottom 25%	1.590-0.101	403(13.78)	1812(5.54)	499(9.15)	635(8.49)	965(15.20)	196(13.87)

**Table 3:** **Distribution of publications from each region in the four grades (2009–2013)**

**Rank**	**IF range**	**China (%)**	**USA (%)**	**UK (%)**	**Germany (%)**	**Japan (%)**	**South Korea (%)**
Top 25%	11.089-4.046	579(27.07)	8705(50.56)	1468(54.29)	1740(45.85)	922(31.50)	250(28.51)
Top 25%-50%	3.693-2.462	669(31.28)	5662(32.89)	659(24.37)	958(25.24)	637(21.76)	246(28.05)
Bottom25%-50%	2.427-1.679	575(26.88)	1890(10.98)	358(13.24)	782(20.61)	902(30.82)	259(29.53)
Bottom 25%	1.590-0.101	316(14.77)	959(5.57)	219(8.10)	315(8.30)	466(15.92)	122(13.91)

### Citation reports

**Figure 3:**
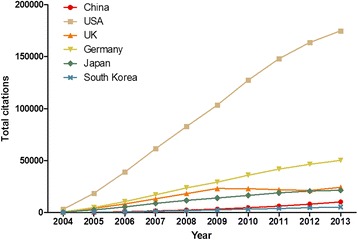
**Total number of citations of articles from each region published in hematology journals between 2004 and 2013.**

The rank of total citations-to-published articles between 2004 and 2013 was as follows: USA (921,553 citations and 93,548 articles); Germany (260,570 citations and 23,970 articles); UK (158,171 citations and 19,393 articles); Japan (120,431 citations and 13,984 articles); China (37,712 citations and 6,666 articles) and South Korea (24,134 citations and 3,694 articles) [Figure 3]. Germany had the highest average citations, meanwhile China had the lowest average citations. It is noteworthy that the average number of citations in Japan surpassed UK and was much higher than South Korea and China [Table 4].

**Table 4:** **Total citations and average citations in the six countries from 2004 to 2013**

**Citations**	**China**	**USA**	**UK**	**Germany**	**Japan**	**South Korea**
	**6666articles**	**93548articles**	**19393articles**	**23970articles**	**13984articles**	**3694articles**
2004	46	3085	642	772	462	39
2005	362	18571	4023	4815	2570	289
2006	900	39048	8372	10611	5387	767
2007	1601	61372	13263	17048	8831	1283
2008	2415	82679	18214	23775	11687	1833
2009	3375	103407	23123	29191	13898	2534
2010	4665	127350	22889	35968	16569	3317
2011	6151	147856	21966	41725	18895	3997
2012	8105	163462	21297	46463	20681	4767
2013	10092	174723	24382	50202	21451	5308
Total	37712	921553	158171	260570	120431	24134
Average	5.657	9.851	8.156	10.871	8.612	6.533

### Top 10 hematology journals

The number of articles from each country of the journals which ranked in the top 10 according to the *JCR 2014* is shown in Tables 5 and 6. The *Blood Review* is a review journal and was not included in the top10. A total of 23,153 articles were published in the top 10 journals by the 6 counties between 2004 and 2013 [Table 5]. Among the 6 countries, USA accounted for the overwhelming majority of the number of articles (14,507/23,153 [62.66%]), while the number of articles contributed by South Korea authors was relatively small (303/23,153 [1.31%]). The number of articles published by Chinese authors was also small (544/23,153 [2.35%]).

**Table 5:** **Number of publications in the top 10 hematology journals from each region (2004–2013)**

**Rank**	**Journal**	**2013IF**	**China (%)**	**USA (%)**	**UK (%)**	**Germany (%)**	**Japan (%)**	**South Korea (%)**	**Total**
1	*Circulation Research*	11.089	44(1.90)	1725(74.68)	110(4.76)	263(11.39)	143(6.19)	25(1.08)	2310
2	*Blood*	9.775	127(1.49)	6342(74.59)	499(5.87)	964(11.34)	508(5.97)	63(0.74)	8503
3	*Leukemia*	9.379	45(3.46)	673(51.81)	142(10.93)	303(23.33)	125(9.62)	11(0.85)	1299
4	*Stem Cells*	7.133	77(4.78)	980(60.79)	96(5.96)	158(9.80)	212(13.15)	89(5.52)	1612
5	*Haematologica*	5.868	36(4.34)	276(33.29)	161(19.42)	269(32.45)	74(8.93)	13(1.57)	829
6	*Thrombosis and Haemostasis*	5.76	49(3.92)	466(37.25)	163(13.03)	445(35.57)	115(9.19)	13(1.04)	1251
7	*Journal of Thrombosis and Haemostasis*	5.55	29(1.93)	863(57.53)	341(22.73)	127(8.47)	130(8.67)	10(0.67)	1500
8	*Arteriosclerosis, Thrombosis, and Vascular Biology*	5.533	72(3.15)	1433(62.77)	156(6.83)	254(11.13)	320(14.02)	48(2.10)	2283
9	*Journal of Cerebral Blood Flow and Metabolism*	5.339	39(2.92)	850(63.67)	150(11.24)	157(11.76)	126(9.44)	13(0.97)	1335
10	*British Journal of Haematology*	4.959	26(1.17)	899(40.30)	845(37.88)	246(11.03)	197(8.83)	18(0.81)	2231
Total			544(2.35)	14507(62.66)	2663(11.50)	3186(13.76)	1950(8.42)	303(1.31)	23153

**Table 6:** **Number of publications in the top 10 hematology journals from each region (2009–2013)**

**Rank**	**Journal**	**2013IF**	**China (%)**	**USA (%)**	**UK (%)**	**Germany (%)**	**Japan (%)**	**South Korea (%)**	**Total**
1	*Circulation Research*	11.089	38(3.45)	846(76.84)	31(2.82)	115(10.45)	60(5.45)	11(1.00)	1101
2	*Blood*	9.775	78(1.72)	3424(75.42)	274(6.04)	494(10.88)	237(5.22)	33(0.73)	4540
3	*Leukemia*	9.379	21(3.23)	359(55.23)	69(10.62)	139(21.38)	57(8.77)	5(0.77)	650
4	*Stem Cells*	7.133	56(7.38)	483(63.64)	40(5.27)	62(8.17)	71(9.35)	47(6.19)	759
5	*Haematologica*	5.868	16(3.49)	172(37.47)	102(22.22)	140(30.50)	24(5.23)	5(1.09)	459
6	*Thrombosis and Haemostasis*	5.76	30(5.18)	216(37.31)	85(14.68)	190(32.82)	47(8.12)	11(1.90)	579
7	*Journal of Thrombosis and Haemostasis*	5.55	19(2.75)	375(54.19)	168(24.28)	69(9.97)	52(7.51)	9(1.30)	692
8	*Arteriosclerosis, Thrombosis, and Vascular Biology*	5.533	57(5.02)	759(66.87)	65(5.73)	110(9.69)	115(10.13)	29(2.56)	1135
9	*Journal of Cerebral Blood Flow and Metabolism*	5.339	27(3.65)	440(59.46)	95(12.84)	100(13.51)	70(9.46)	8(1.08)	740
10	*British Journal of Haematology*	4.959	18(1.72)	444(42.41)	397(37.92)	109(10.41)	72(6.88)	7(0.67)	1047
Total			360(3.08)	7518(64.25)	1326(11.33)	1528(13.06)	805(6.88)	165(1.41)	11702

When comparing the data from 2009–2013 [Table 6], the percentage of articles in the top 10 journals from UK, Germany, and Japan decreased slightly, while the percentage of articles in the top 10 journals from China, USA, and South Korea had a small rise; all of these changes were no more than 2 percentage points.

### Most published journals

The journals in which articles from the six countries were published most frequently are listed in Tables 7 and 8. It is worth noting that the same journal (*Blood*) was the most frequent target journal in the USA and Germany; *Blood* is one of the most influential journals in the field of hematology.

**Table 7:** **Ten most published hematology journals in each region (2004–2013)**

**Rank**	**China**	**N**	**USA**	**N**	**UK**	**N**	**Germany**	**N**	**Japan**	**N**	**South Korea**	**N**
1	*LR*	214	*Blood*	6342	*BJH*	845	*Blood*	964	*IJH*	1020	*AH*	114
2	*LL*	188	*CR*	1725	*Blood*	499	*TH*	445	*Blood*	508	*SC*	89
3	*IJH*	168	*PBC*	1678	*JTH*	341	*Leukemia*	303	*TAD*	445	*SCD*	79
4	*TR*	141	*ATVB*	1433	*Haemophilia*	189	*AH*	299	*ATVB*	320	*IJH*	78
5	*SCD*	133	*Transfusion*	1351	*VS*	188	*CHM*	272	*LR*	235	*Acta Heamatol*	73
6	*Blood*	127	*BBMT*	1173	*TH*	163	*Haematologica*	269	*TR*	219	*BMT*	67
7	*Shock*	119	JLB	1048	*Haematologica*	161	*CR*	263	*SC*	212	*LR*	63
8	*Cytotherapy*	111	*LL*	1028	*ATVB*	156	*Transfusion*	255	*BJH*	197	*Blood*	63
9	*AH*	106	*SC*	980	*JCBFM*	150	*ATVB*	254	*BMT*	195	*LL*	56
10	*JHO*	79	*AJH*	899	*LL*	149	*BJH*	246	*EJH*	182	*TR*	53

**Table 8:** **Ten most published hematology journals in each region (2009–2013)**

**Rank**	**China**	**N**	**USA**	**N**	**UK**	**N**	**Germany**	**N**	**Japan**	**N**	**South Korea**	**N**
1	*LR*	152	*Blood*	3424	*BJH*	397	*Blood*	494	*IJH*	547	*AH*	82
2	*LL*	143	*PBC*	1006	*Blood*	274	*CHM*	197	*Blood*	237	*SCD*	58
3	*IJH*	123	*CR*	846	*JTH*	168	*TH*	190	*TAD*	201	*IJH*	51
4	*TR*	112	*ATVB*	759	*Haematologica*	102	*AH*	144	*TR*	120	*SC*	47
5	*SCD*	108	*BBMT*	710	*Haemophilia*	100	*Haematologica*	140	*ATVB*	115	*LR*	43
6	*AH*	89	*Transfusion*	709	*JCBFM*	95	*Leukemia*	139	*LR*	109	*Acta Haematol*	42
7	*Shock*	80	*LL*	573	*TH*	85	*TMH*	136	*BMT*	92	*Blood*	33
8	*Blood*	78	*AJH*	492	*AJH*	73	*CR*	115	*EJH*	74	*TR*	33
9	*JHO*	78	*SC*	483	*Leukemia*	69	*Transfusion*	113	*BJH*	72	*IJLH*	32
10	*Cytoherapy*	75	*BJH*	444	*Transfusion*	68	*ATVB*	110	*SC*	71	*ATVB*	29

*Acta Haematol*, IF = 0.994; AJH, *Am J Hematol*, IF = 3.477; AH, *Ann Hematol*, IF = 2.396; ATVB, *ArteriosclerThrombVasc Biol*, IF = 5.533; BBMT, *Biol Blood Marrow Transplant*, IF = 3.348; *Blood*, IF = 9.775; BP, *Blood Purif*, IF = 1.92; BMT, *Bone Marrow Transplant*, IF = 3.466; BJH, *Br J Haematol*, IF = 4.959; CR, *Circ Res*, IF = 11.089; *Cytotherapy*, IF = 3.1; CHM, *Clin Hemorheol Micro*, IF = 2.215; EJH, *Eur J Haematol*, IF = 2.414; *Haematologica*, IF = 5.868; *Haemophilia*, IF = 2.468; IJH, *Int J Hematol*, IF = 1.679; IJLH, *Int J Lab Hematol,* IF = 1.87; JCBFM, *J Cerebr Blood F Met*, IF = 5.339; JHO, *J Hematol Oncol*, IF = 4.933; JLB, *J Leukoc Biol*, IF = 4.304; JPHO, *J Pediatr Hematol Oncol*, IF = 0.956; JTH, *J Thromb Haemost*, IF = 5.55; *Leukemia*, IF = 9.379; LL, *Leuk Lymphoma*, IF = 2.605; LR, *Leuk Res*, IF = 2.692; PBC, *Pediatr Blood Cancer*, IF = 2.562; *Shock*, IF = 2.732; SC, *Stem Cells*, IF = 7.133; SCD, *Stem Cells Dev,* IF = 4.202; TAD, *Ther Apher Dial*, IF = 1.532; TH, *Thromb Haemost*, IF = 5.76; TMH, *Transfus Med Hemoth*, IF = 2.011; TR, *Thromb Res*, IF = 2.427; *Transfusion*, IF = 3.568; VS, *Vox Sang*, IF = 3.303.

Articles from China were most often published in *Leukemia Research*, while the *International Journal of hematology* and *annals of hematology* were the most published journals in Japan and South Korea, respectively. These three journals were not amongst the top 10 journals [Tables 7 and 8]. When comparing the results between 2004–2013 and 2009–2013, there was no significant difference.

## Discussion

This is the first study that compared the quantity and quality of publications in hematology-related journals from China and the USA, UK, Germany, Japan, and South Korea. Publications from the six countries were extracted based on the PubMed database, a free search engine primarily accessing the MEDLINE database of references and abstracts pertaining to the life sciences and biomedical topics. The PubMed database was created by the United States National Library of Medicine (NLM) at the National Institutes of Health. The IF of each journal was calculated using the *JCR 2014*, which helps to quantify the influence of research and impact on the journal or category levels; the IF also indicates the relationship between citing and cited journals.

As shown in Figure 1, only China and South Korea exhibited a sustained trend in growth between 2004 and 2013 with respect to the number of publications in hematology-related journals. The USA had an upward trend in the number of hematology articles published between 2004 and 2011, but the trend was reversed in the last 3 years. The number of articles from the other three countries (the UK, Japan, and Germany) remained relatively stable. In 2013, China surpassed the UK and approached Japan with respect to the number of hematology articles published. There is no doubt that the USA is the leader in this area of research and has a much stronger research effort than the other five countries. The same pattern was demonstrated when the publication of different types of articles was analyzed. The USA was the leader in publishing each type of article, especially reviews and RCTs. A review is an attempt to summarize the current state of understanding on a specific topic and make comments, and is usually written by a leading expert in the area [5]. A RCT is the gold standard for a clinical trial, and is often used to determine the effectiveness of various types of medical interventions. A RCT may provide information about adverse effects, such as drug reactions. A RCT is considered to be the most reliable form of scientific evidence in the hierarchy of evidence, and influences healthcare policy and practice. The results herein demonstrated that the USA has made the greatest contribution in the field of hematology. Specially, the number of meta-analyses published from China approached the number of meta-analyses published from the USA, which can be explained by the rapid development of evidence-based medicine in China.

With respect to the number of articles, China has made tremendous progress. The GDP of China ranks second in the world behind the USA. Between 2000 and 2012, the amount of research spent in China increased from $10.8 billion to $168 billion. According to an OECD estimation, the Chinese government invested 1.98% of the GDP in research in 2012 [6], and the amount announced by the government may reach 2.5% in 2020 [7]. Various types of medical research-funded projects also increased considerably in the past decade. For example, the Department of Health Sciences affiliated with the National Natural Science Foundation of China was founded in 2009 and there is a special office dedicated to funding respiratory, hematology, and circulatory system research. In fact, funds from this office increased significantly from 228.81 million yuan in 2010 to 591.09 million yuan in 2013. Furthermore over the past decade, the number of Chinese scientific and technical personnel experienced the same rapid growth. All of those factors have greatly promoted Chinese research in the field of hematology.

The IF of an academic journal is a measure reflecting the average number of citations to recent articles published in the journal. The IF is frequently used as a proxy for the relative importance of a journal within its field; journals with higher IFs are deemed to be more important than journals with lower IFs. Despite considerable criticism regarding the IF, the IF is universally accepted as the most convincing index for quality evaluation of journals [8,9]. In the past 10 years, the cumulative IFs of articles published in the USA were several times higher than the other countries. With respect to the median IF of published articles, the UK led the countries surveyed, while the articles published from Japan, China, and South Korea were significantly lower than articles published from Europe and USA; these findings did not change significantly in 2009–2013. When comparing the distribution of publications related to the IF, articles published from Europe and USA were shown to be mostly published in the top 25% of journals; however, China lagged behind and ranked last with respect to publications in the top 25% of journals. This is an important method regarding the evaluation of publication quality, and the result may alert Chinese scientists in this regard. This phenomenon, at least in part, may be a reflection of China, Japan, and South Korea being non-English speaking countries. Indeed, European and USA researchers may not pay close attention to scientific findings published in languages other than English.

Despite the rapid growth in the number of articles from China, the quality was not satisfactory. This may be associated with the current imperfect evaluation system on research performance that exists in mainland China [10,11], which enforced researchers to pay more attention to quantity rather than quality. The citation reports and the number of publications in the top 10 journals further support the above conclusions.

There were several limitations in the present study. Even though the journal list was generated based on the hematology category in SCIE subject categories, there may be excellent hematology journals that were not included by SCIE. Second, the publication of some international collaborative studies may have only listed one address for all authors. Therefore, when included in this study, the contributions of other countries were ignored. Third, there are some hematology-related articles published in general medical journals, which likely affected our results.

## Conclusions

In conclusion, there is no doubt that the USA has played the most important role in the field of hematology research in the past decade. Overall, the quality of publications in European countries is better than Asian countries. We are delighted to see that China is closing the gap with the developed countries. With respect to future research, scientists from mainland China should take effective measures and strive to improve the quality of research.

## Methods

### Search strategy

Sixty-eight hematology journals were selected from the Hematology category of the Science Citation Index Expanded (SCIE) subject categories, as designated by Thomson Reuters. The 68 journals, which are all indexed by PubMed, cover resources that involve blood and blood-forming tissues, as well as the function, diseases, and treatments of the hematologic system. The topics included hemophilia, neoplastic disorders of the blood or lymphoid tissues, and mechanisms and disorders of thrombosis. A computerized literature search was conducted in the PubMed database on 2 October 2014, and the articles published in the 68 journals from China, the USA, the UK, Germany, Japan, and South Korea between 1 January 2004 and 31 December 2013 were identified.

The ISSN (print) and publication date (print) were used to perform searches in the PubMed database. The search terms were “China[ad] not Hongkong[ad] not Taiwan[ad]”, “USA[ad]”, “UK[ad]”, “Germany[ad]”, “Japan[ad]”, “Korea[ad]” and “0001-5792 OR 0361–8609 OR 0939–5555 OR 1079–5642 OR 1521–6926 OR 1083–8791 OR 0006-355X OR 0006–4971 OR 1079–9796 OR 0957–5235 OR 0253–5068 OR 0268-960X OR 1723–2007 OR 0268–3369 OR 0007–1048 OR 0009–7330 OR 1076–0296 OR 1386–0291 OR 2152–2650 OR 1040–8428 OR 1558–8211 OR 1065–6251 OR 1465–3249 OR 0902–4441 OR 0301-472X OR 1747–4086 OR 0234–5730 OR 0390–6078 OR 1351–8216 OR 0720–9355 OR 0278–0232 OR 1024–5332 OR 1520–4391 OR 0889–8588 OR 0363–0269 OR 0971–4502 OR 0925–5710 OR 1751–5521 OR 0271-678X OR 0733–2459 OR 1756–8722 OR 0741–5400 OR 1077–4114 OR 1538–7933 OR 0929–5305 OR 0887–6924 OR 1042–8194 OR 0145–2126 OR 1073–9688 OR 1545–5009 OR 0888–0018 OR 0953–7104 OR 0037–1963 OR 0094–6176 OR 1073–2322 OR 1066–5099 OR 1547–3287 OR 1744–9979 OR 0340–6245 OR 0049–3848 OR 0041–1132 OR 1473–0502 OR 1246–7820 OR 1660–3796 OR 0958–7578 OR 0887–7963 OR 1300–7777 OR 0042–9007.” Articles in which the first author was affiliated with the above geographic regions were considered to be research output from the respective region. According to the publication types in PubMed, the number of clinical trials, randomized controlled trials (RCTs), meta-analyses, and reviews from each geographic region were extracted.

We used three methods to assess the quality of the literature. First, the cumulative impact factors (IFs) and the median IF were calculated using *Journal Citation Reports (JCR) 2014*, which is published by Thomson Reuters, and the article distribution related to IFs was also assessed for each region. Second, citation reports of articles showing an affiliation with each region were collected using the Web of Science. Third, articles published in the top 10 hematology journals (based on IFs) were counted, while the 10 most published hematology journals in each region, according to the publication number, were also identified. The above comparisons were also conducted based on the data from 2009–2013 (Additional file 1).

### Statistical analysis

We focused on the trends and not to test the hypothesis regarding the relative contributions of each region. Therefore, only simple descriptive statistics (e.g., sum and median) are provided in this study.

## Additional file

Additional file 1:
**The main flowchart of the study.**
