# Supplementary material for: Hematology research output from Chinese authors and other countries: a 10-year survey of the literature
Source: J Hematol Oncol. 2015 Feb 6;8:8. doi: 10.1186/s13045-014-0103-3 (PMC4332745; doi:10.1186/s13045-014-0103-3)
Supplement: Additional file 1: — The main flowchart of the study. [file 13045_2014_103_MOESM1_ESM.docx]

**Flowchart**

| Search in PUBMED database.  Search strategy was described in the method part. |
| --- |

| Inclusion criteria:  Time limited :2004 to 2013 |
| --- |

| The total number of hematology-related articles: 120,641 |
| --- |

Identity the number of articles from the six countries(China, USA, UK, Germany, Japan and South Korea)

| \| China  2,924 \| USA  32,732 \| UK  5,453 \| Germany  7,479 \| Japan  6,347 \| South Korea 1,413 \|  \| \| --- \| --- \| --- \| --- \| --- \| --- \| --- \| |
| --- | --- | --- | --- | --- | --- | --- | --- |

| Discussed the contribution to hematology research from each region and the gap between China and others |
| --- |

| Evaluated the quantity and quality of the articles form each region based on the approach mentioned above |
| --- |

| Search the Impact factor of articles from the above-mentioned regions, besides the publication type, numbers in TOP10 journals and most published journals |
| --- |

citation reports of articles from each region were collected using the Web of Science
